# Mono-(2-Ethylhexyl) Phthalate Induces Injury in Human Umbilical Vein Endothelial Cells

**DOI:** 10.1371/journal.pone.0097607

**Published:** 2014-05-16

**Authors:** Jin-Bao Ban, Xiao-Wu Fan, Qi Huang, Bin-Feng Li, Chen Chen, Hua-Chuan Zhang, Shun-Qing Xu

**Affiliations:** 1 Department of General Thoracic Surgery, Tongji Hospital Attached to Tongji Medical College, Huazhong University of Science and Technology, Wuhan, Hubei, People's Republic of China; 2 Key Laboratory of Environment and Health, School of Public Health, Tongji Medical College, Huazhong University of Science and Technology, Wuhan, China; University of Sassari, Italy

## Abstract

Mono-(2-ethylhexyl) phthalate (MEHP), the active metabolite of di-(2-ethylhexyl) phthalate (DEHP), is a widespread environmental contaminant and has been proved to have potential adverse effects on the reproductive system, carcinogenicity, liver, kidney and developmental toxicities. However, the effect of MEHP on vascular system remains unclear. The main purpose of this study was to evaluate the cytotoxic effects of MEHP on human umbilical endothelial cells (HUVEC) and its possible molecular mechanism. HUVEC cells were treated with MEHP (0, 6.25, 12.5, 25,50 and 100 µM), and the cellular apoptosis and mitochondrial membrane potential as well as intracellular reactive oxygen species were determined. In present study, MEHP induced a dose-dependent cell injury in HUVEC cell via an apoptosis pathway as characterized by increased percentage of sub-G1, activation of caspase-3, -8and -9, and increased ratio of Bax/bcl-2 mRNA and protein expression as well as cytochrome C releasing. In addition, there was obvious oxidative stress, represented by decreased glutathione level, increased malondialdehyde level and superoxide dismutase activity. N-Acetylcysteine, as an antioxidant that is a direct reactive oxygen species scavenger, could effectively block MEHP-induced reactive oxygen species generation, mitochondrial membrane potential loss and cell apoptosis. These data indicated that MEHP induced apoptosis in HUVEC cells through a reactive oxygen species-mediated mitochondria-dependent pathway.

## Introduction

Phthalates are common synthetic plasticizers used in polyvinyl chloride (PVC) industry, including packaging materials, cosmetics, clothing, toys and medical devices [1] and the di-(2-ethylhexyl) phthalate (DEHP) is mostly used, especially in China [2]. Because of uncovalent bounding to polymers in plastics, DEHP is gradually released from polymer matrices to environment and becomes a widespread environmental pollutant [3]. Human beings are exposed to DEHP predominantly via food wrap, cosmetics, industrial products as well as medical devices [4]. MEHP is the active monoester metabolite of DEHP in vivo and discharged in the urine [5]. It has been reported that in the NHANES cohort between 1999 and 2006, MEHP was found in their urine samples in about 80% participants [6], which indicated that the human DEHP exposure was universal.

As an endocrine disruptor, DEHP altered male sexual differentiation [7] and disturbed energy metabolism in animals [[8],[9]]. Investigations mainly focused on the potential adverse effects of DEHP and MEHP, its active metabolite, on the reproductive system, carcinogenicity, liver, kidney and developmental toxicities [10], [11]. However, reports about the DEHP and/or MEHP induced injury of cardiovascular system are rare. The previous work in our lab has demonstrated that the maternal DEHP exposure throughout gestation and lactation disturbed the nephron formation and induced glomerulosclerosis in offspring [12], indicating that the DEHP or its active metabolite MEHP may have adverse effects on endothelial cells. Therefore, the present study investigated the MEHP induced cytotoxicity in human umbilical endothelial cells (HUVEC) and the potential mechanisms.

## Materials and Methods

### Material

We purchased MEHP from Accustandard (New Haven, CT). Dimethyl sulfoxide (DMSO), N-acetylcysteine (NAC) and 3-(4,5-dimethylthiazol-2-yl)-2,5-diphenyltetrazoliumbromide (MTT), were obtained from Sigma (St Louis, MO, USA). Ribonuclease A, Triton X-100, Propidiumiodide (PI) and 2,7-dichlorofluoroscein diacetate (DCFH-DA) were purchased from Amresco (Cochran Solon, OH, USA). RPMI 1640 cell culture medium, penicillin, streptomycin, heat-inactivated fetal bovine serums were purchased from invitrogen (Carlsbad, CA, USA). SV Total RNA Isolation System was purchased from Promega (Madsion, WI, USA). M-MuLV Reverse transcriptase was obtained from (Fermentas, Ontario, Canada). The SYBR Green PCR Master Mix was purchased from Applied Biosystems (Foster City, CA, USA). Polyvinylidene difluoride (PVDF) membrane was purchased from Bio-Rad (Hercules, CA, USA). Total superoxide dismutase (SOD) assay kit, glutathione (GSH) assay kit, malondialdehyde (MDA) assay kit, caspase-3, -8 and 9 activity kits, mitochondria/cytoplasm fractionation kit, nuclear and cytoplasmic protein extraction kit, BCA protein assay kit and enhanced chemiluminescence (ECL) were purchased from Beyotime (Shanghai, China). The rabbit polyclonal anti Cytochrome C, B-cell lymphoma 2 (Bcl-2) and Bcl-2-associated X protein (Bax) antibodies, the goat anti rabbit and goat anti mouse IgG antibodies and the cell counting kit-8 (CCK-8) were purchased from Boster (Wuhan, Hubei, China). The mouse monoclonal anti β-actin antibodies was purchased from Tianjin Sungene Biotech Co. (Tianjin, China).

### Cell Culture and Treatment

Human umbilical vein endothelial cells (HUVEC) were purchased from China Center of Type Culture Collection (CCTCC) in Wuhan. The cells were cultured in RPMI 1640 medium which had been complemented with 10% heat-inactivated fetal bovine serum, penicillin (100 IU/ml), streptomycin (100 mg/ml) and 2 mM L-glutamine in a humidified CO_2_ incubator with 5% CO_2_ at 37°C MEHP was dissolved in DMSO as stock solutions. The MEHP work solutions (6.25, 12.5, 50, or 100 µM) were made immediately prior to the administration. The DMSO concentration was 0.1% for all treatment groups. Control cells were treated with 0.1% DMSO only.

### Assessment of Cell Viability

The Cell Counting Kit-8 (CCK-8) was utilized to assess cell viability. The HUVEC cells (0.2×10^4^ per well) were plated into 96-well plates for 24 hours. Then, various concentrations of MEHP (6.25, 12.5, 50 and 100 µM) were added. 24 hours and 48 hours later, CCK solutions (10 µl per well) was added into the 96-well plates. The cells were incubated for 4 hours at 37°C. The optical density value at 490 nm was measured by a microplate reader. The percentage of treatment to control optical density represented cell viability. Each measurement was carried out in triplicate.

### Detection of apoptosis

FACScan Flow Cytometer (BD Biosciences) was used for quantifying Apoptotic cells by determining DNA content of cells. According to the reports of Nicoletti et al., PI staining was utilized [13]. The DNA staining solution contains 50 mg/ml PI, 0.1 mM EDTA (pH 7.4), 0.1% tritonX-100 and 50 mg/ml RNase. HUVEC cells (2×105 per well) were cultured in six-well plates for 24 hours, and treated with various concentrations of MEHP (0, 6.25, 12.5, 25, 50 and 100 µM)for another 24 hours. After Trypsin/EDTA digestion (37°C, 5 min) and phosphate-buffered saline (PBS) washing, the HUVEC cells were collected. Both control and treated cells were treated with ice-cold 70% (v/v) and kept overnight at 4°C to get the cells fixed and permeabilized. To thoroughly removed ethanol, the overnight treated cells were centrifuged and washed with PBS. Then HUVEC cells were re-suspended in 400 ml DNA staining solution for 40 min at 37°C in the dark. The PI fluorescence (FL-2 filter; 585 nm) was detected by flow cytometer mentioned above. The cell apoptosis rate is represented by the percentage of cells with hypodiploid DNA contents. Each measurement was carried out in triplicate.

### Measurement of MDA, GSH content and SOD activity

The HUVEC cells (2×10^5^ per well) were cultured in six-well plates for 24 hours, and incubated with MEHP of various concentrations (0, 6.25, 12.5, 25, 50 and 100 µM) for another 24 hours. The levels of lipid peroxidation in HUVEC cells was assessed by MDA assay kits according to the manufacture's protocol. The GSH level was measured by GSH assay kits according to the manufacture's protocol. The activity of SOD of HUVEC cells was detected by SOD assay kit according to the manufacture's protocol. Both MDA and GSH levels were represented in nmol/mg protein, while the SOD activity was U/mg protein. Each measurement was carried out in triplicate.

### ROS measurement

According to the report of Robinson et al. [14], we assessed intracellular ROS levels by DCFH-DA to investigate whether the MEHP exposure induces oxidative stress in HUVEC cells. The fluorescence intensity is corresponding to the ROS generation in the treated cells. HUVEC cells (1×10^4^ per well) were plated into 48-well plates for 24 hours, and incubated with MEHP of various concentrations (0, 6.25, 12.5, 25, 50 and 100 µM) for another 24 hours. Then 10 mM DCFH-DA was supplemented and the cells were incubated at 37°C for 20 min. When washed three times with PBS, the plates were immediately inserted into an Olympus CKX41-F32FL fluorescence microscope to detect and photograph the fluorescence.

### Mitochondrial Membrane Potential Assay

We used the JC-1 Detection Kit in order to investigate the mitochondrial membrane potential (MMP) changes induced by MEHP in HUVEC cells according to the manufacturer's protocol. JC-1 is a kind of fluorescent carbocyanine dye. Depend on the status of MMP, JC-1 may assemble on the mitochondrial membrane in monomer forms or dimer forms. When the mitochondrial membrane is highly polarized, JC-1 becomes dimmers and transmits red fluorescence. If the mitochondrial membrane is depolarizeded, JC-1 converts into monomers and transmits green fluorescence. Therefore, the green fluorescence represents the MMP loss. HUVEC cells (1×104 per well) were cultured in 48-well plates for 24 hours, and incubated with MEHP of various concentrations (0, 6.25, 12.5, 25, 50 and 100 µM) for another 24 hours. Controls were treated with DMSO only. After incubated with JC-1 for 30 min at 37°C in the dark, the treated cells were immediately photographed by the Olympus CKX41-F32FL fluorescence microscope.

### Caspase-like Activity Analysis

To estimate the activities of caspase-3, -8 and -9 in MEHP treated HUVEC cell, we used caspase-3, -8 and-9 activity kits according to the manufacturer's instruction. After treated with MEHP in various concentrations (0, 6.25, 12.5, 25, 50 and 100 µM) for 24 hours, the HUVEC cells were blended with 100 ml lysate and then centrifuged at 16,000 g for 10 min at 4°C. The supernatant were incubated with caspase substrates at 37°C for 2 hours. Finally the absorbance of the supernatant was detected by a microplate reader at 405 nm. Bradford method was used in total protein concentration were measurement of supernatants. Each measurement was carried out in triplicate.

### RNA Extraction, Reverse Transcription and Real-time PCR

The relative gene mRNA expression of B-cell lymphoma 2 (Bcl-2) and Bcl-2-associated X protein (Bax) in treated HUVEC cells was analyzed by Real-time PCR (QPCR). SV Total RNA Isolation System was utilized in total RNA extraction according to the manufacturer's protocol. The RNA concentrations were confirmed by absorbance at 260 nm. The purity of RNA was confirmed by the ratio the optical densities at 260 and 280 nm. M-MuLV Reverse transcriptase was used in reverse transcription for cDNA synthesis according to the manufacture's protocol. GAPDH was used as an endogenous reference gene. The primers for target genes were obtained from the NCBI GeneBank database. The SYBR Green PCR Master Mix was used in QPCR. The Applied Biosystems model 7900HT Fast Real-Time PCR System runs the reaction. The cycling programs were as follows: 95°C for 10 min, followed by 40 cycles of 95°C for 15 s and 60°C for 1 min. The fluorescence signal was detected during the extension step in each cycle. Normalized to GADPH, 2^−ΔΔCT^ method was used in calculation of target gene expression. The result was represented in a relative value compared to the control. Each measurement was carried out in triplicate.

The primers of target genes was as follows: Bcl-2 (Genbank Accession no. NM_000657) forward primer: 5′ -AGG AAG TGA ACA TTT CGG TGA C-3′ and reverse primer: 5′-GCTCAG TTC CAG GAC CAG GC-3′. Bax (Genbank Accession no. NM_138763) forward primer: 5′-TGC TTC AGG GTT TCA TCC AG-3′ and reverse primer: 5′-GGC GGC AAT CAT CCT CTG-3′.

### Western blotting for Cytochrome C, B-cell lymphoma 2 (Bcl-2) and Bcl-2-associated X protein (Bax)

The release of mitochondrial cytochrome C and expression of Bcl-2 and Bax was detected by western blot. The nuclear and cytoplasmic protein extraction kit was used in protein extraction BCA protein assay kit was used in protein concentration measurement according to the manufacture's instruction. The sodium dodecyl sulfate polyacrylamide gel (SDS-PAGE) electrophoresis separated the proteins. Then the proteins transferred onto PVDF membrane. The blockage buffer, 5% non-fat milk powder dissolved in Tris buffered saline-Tween 20 (TBST, Ph 7.4), was used in membrane incubating for 1–2 hours to reduce nonspecific bindings. Specific primary antibodies of each target protein were used in membrane incubating at 4°C overnight. In order to eliminate unbinding primary antibodies, the membrane was washed 3 times with TBST for 15min, and then incubated with secondary antibodies, which were conjugated by the horseradish peroxidase (HRP), for 4 hours. ECL was used in protein visualization according to the manufacturer's instructions. The β-actin was the endogenous reference protein. Each measurement was carried out in triplicate.

### Effects of antioxidant on MEHP-induced Cytotoxicity

NAC, dissolved in DMSO, treated the HUVEC cells for 1 hour prior to MEHP administration. Then the HUVEC cells were incubbated with MEHP for 24 hours. In order to exclude the direct reaction to MEHP, which may scavenge MEHP, the cells were washed with PBS before MEHP administration. In control group, cells were treated with 0.1% DMSO only. The NAC experiment carried out in MTT assay, cell apoptosis assessment, ROS generation assay, MMP assay, Real-Time PCR and Western-Blot.

### Statistical Analysis

All data were represented in form of mean±SEM. SPSS 13.0 software (SPSS, Inc., Chicago, IL, USA) was used in statistical analysis. Comparison between groups was performed by using one-way ANOVA followed by least significant difference (LSD) and Dunnett's T3 test. *P*<0.05 was considered to be statistically significant in all experiments.

## Results

### MEHP reduces cell viability and induces apoptosis in HUVEC cells

**Figure 1:**
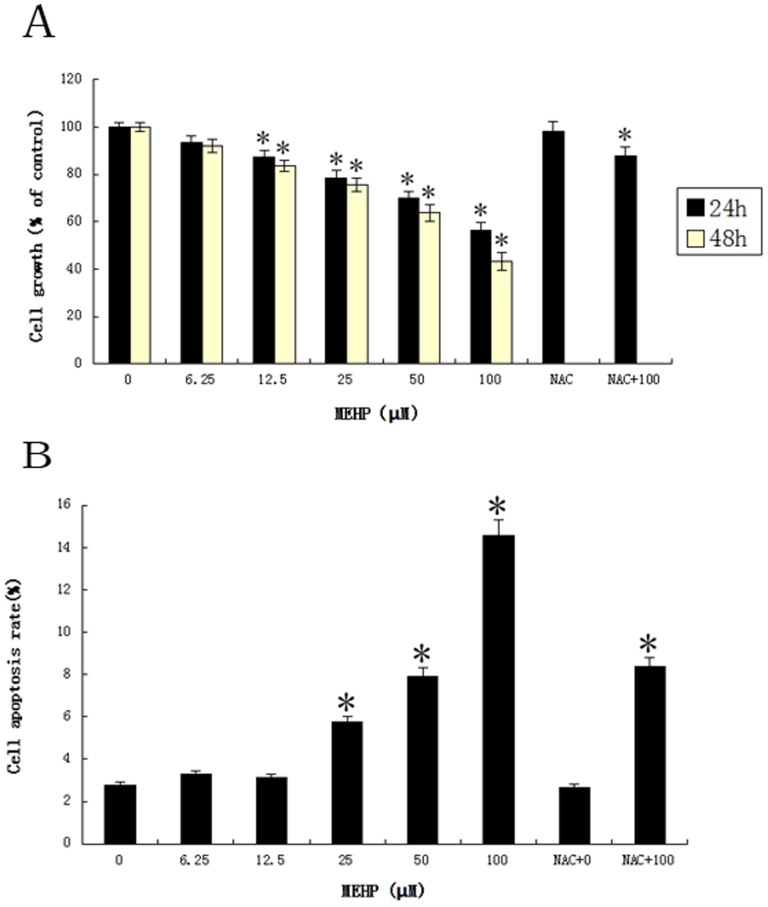
MEHP attenuated the viability of HUVEC cells. In MEHP treatment group, the HUVEC cells were treated with MEHP (0, 6.25, 12.5, 25, 50 and 100 µM) for 24 and 48 hours. In NAC+MEHP treatment group, the NAC was dissolved in DMSO, the HUVEC cells was treated with NAC for 1 hour before MEHP treatment, and then the cells were treated with MEHP (0,100 µM) for 24 hours. The cell viability of both groups were measured by cell counting kit-8 (CCK-8). The treatment to control ratio of optic density represents the cell survival. Data from three independent experiment was represented in the form of mean±SEM; n  = 6. * P<0.05 was considered as statistically significant difference compared to the control group. (B) MEHP induced cell apoptosis in HUVEC cells. In MEHP treatment group, the HUVEC cells were treated with MEHP (0, 6.25, 12.5, 25, 50 and 100 µM) for 24 hours. In NAC+MEHP treatment group, the HUVEC cells was treated with NAC for 1 hour before MEHP treatment, and then the cells were treated with MEHP (0,100 µM) for 24 hours. The cell apoptosis rate in both groups was measured by sub-G1 analysis. Data from three independent experiment was presented in the form of mean±SEM; n = 6. * P<0.05 was considered as statistically significant difference compared to control group.

To investigate the effect of MEHP on cell viability, the HUVEC cells were cultured with MEHP (0, 6.25, 12.5, 25, 50 and 100 µM) for 24 and 48 hours. The survival rate was determined by Cell counting kit-8. The Figure 1A shows the MEHP reduced cell viability in both dose- and time-dependent manner. The cell viability started to decrease upon exposing to MEHP above 12. 5 µM. Treatment with 100 µM MEHP for 48 h, the viability of HUVEC cells decreased approximately 50% (Figure 1A.). To further assess the apoptosis induced by MEHP, the cytometric analysis after PI staining was performed. As showed in Figure 1B, treated with MEHP (0, 6.25, 12.5, 25, 50 and 100 µM) for 24 hours, the percentage of apoptotic cells increased in a dose dependent manner. When the MEHP concentration reached 25 µM, the cells in this group slightly showed more cell apoptosis compared to the control group. When it reached 100 µM, the apoptosis rate in this group was quadrupled.

### Intracellular MDA, GSH levels and SOD activities after MEHP treatment

**Figure 2:**
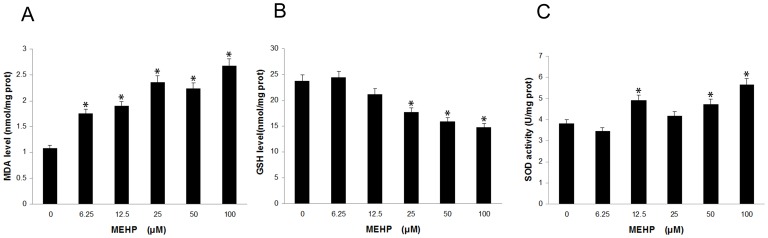
MEHP affected intracellular malondialdehyde (MDA), glutathione (GSH) levels and superoxide dismutase (SOD) activities in HUVEC cells. After treatment with MEHP (0, 6.25, 12.5, 25, 50 and 100 µM) for 24 hours, the MDA (A), GSH (B) levels and SOD activities (C) were measured by corresponding assay kit. Data from three independent experiments was presented in the form of mean±SEM; n = 6. * P<0.05 was considered as statistically significant difference compared to the control group.

The intracellular MDA, GSH and SOD levels of HUVEC cells were detected 24 hours after treated with MEHP (0, 6.25, 12.5, 25, 50 and 100 µM). As showed in Figure 2, the MDA level and the SOD activities increased in a dose dependent way while the GSH levels declined also in a dose dependent manner. The MDA level began to rise at the MEHP concentration of 6.25 µM. When the concentration reached 12.5 µM, the SOD activity increased and the GSH level showed no significant changes until the MEHP concentration reached 25 µM.

### ROS generation in HUVEC cells by MEHP exposure

**Figure 3:**
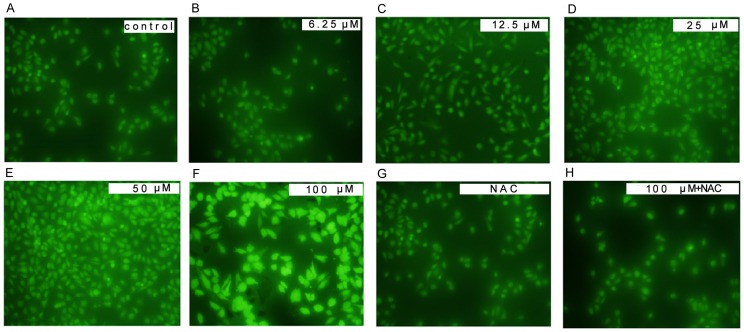
MEHP induced reactive oxygen species (ROS) generation in HUVEC cells. In MEHP treatment group, the HUVEC cells were treated with 0 µM (A), 6.25 µM (B), 12.5 µM (C), 25 µM (D), 50 µM (E) and 100 µM (F) MEHP for 24 hours. In NAC+MEHP treatment group, the HUVEC cells were pretreated for 1 hour before the MEHP treatment and then treated with 0 µM (G) and 100 µM (H) MEHP for 24 hours. After cultured with 10 mM 2,7-dichlorofluoroscein diacetate (DCFH-DA), the HUVEC cells in both groups were photographed by a fluorescence microscope (400x). Data was collected from three independent experiments.

For ROS can induce oxidative modification in macromolecules in cells, which may result in cell apoptosis, the ROS levels were measured by DCF-DA, a ROS sensitive fluorometric probe, to certify the roles of ROS generation in the MEHP-induced HUVEC cell apoptosis. The fluorescence was detected by an Olympus CKX41-F32FL fluorescence microscope after the HUVEC cells treated with MEHP (0-100 µM) for 24 hours. In treatment group, the ROS generation was significantly higher than the control group (Figure 3).

### LOSS of Mitochondrion Membrane Potential (MMP) induced by MEHP

**Figure 4:**
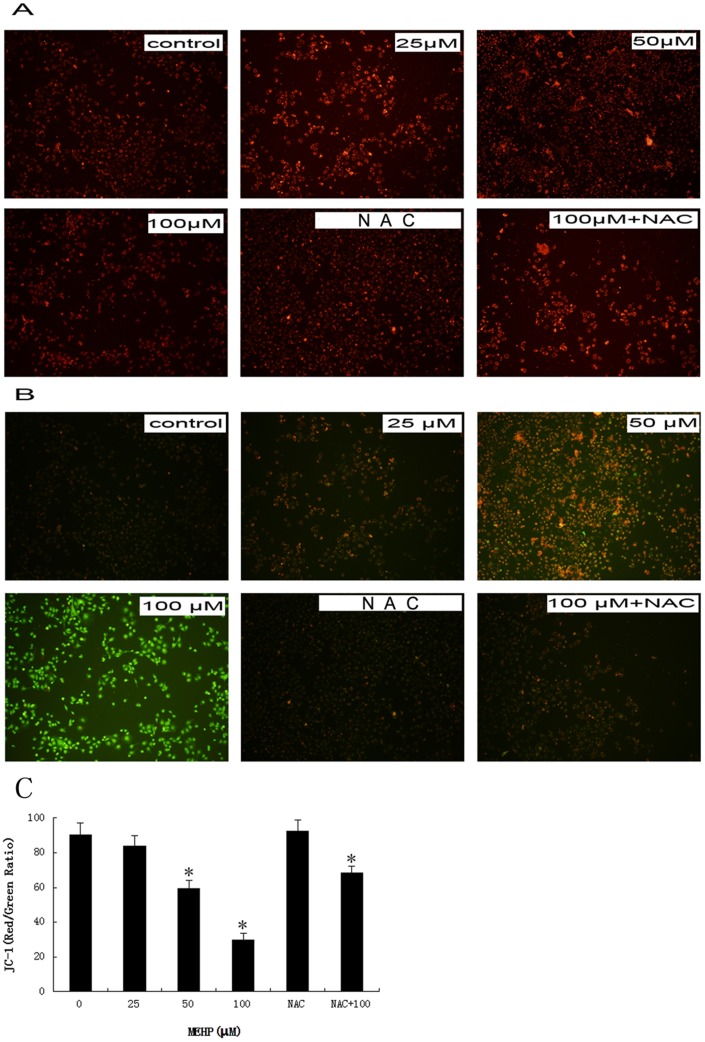
MEHP induced the mitochondrial membrane potential (MMP) loss in HUVEC cells. In MEHP treatment group, the HUVEC cells were treated with 0 µM, 25 µM, 50 µM and 100 µM MEHP for 24 hour. In NAC+MEHP treatment group, the HUVEC cells were pretreated for 1 hour before the MEHP treatment and then treated with 0 µM and 100 µM MEHP for 24 hour. After cultured with JC-1 for 30 minute at 37°C in the dark, the HUVEC cells in both groups were photographed by a fluorescence microscope (100X).Red fluorescence (A) represents mitochondria with intact membrane potential.Green fluorescence (B) represents de-energized mitochondria. The ratio of red fluorescence to green fluorescence was quantified, presented as mean± SEM; n = 3, * P<0.05 was considered as statistically significant difference compared to control group.(C).

It is reported that MMP decrease is associated with mitochondrion dysfunction in cell apoptosis[15]. Therefore, we evaluate the MMP of the MEHP treated HUVEC cells. When treated with MEHP in concentration of 0, 25, 50 and 100 µM for 24 hours, the JC-1 fluorescence was photographed by a fluorescence microscope mentioned above. As shown in Figure 4, the cells of control group showed strong red fluorescence which represents high MMP, whereas the green fluorescence showed increased intensity along with the higher MEHP concentration.

### MEHP induces cell apoptosis of HUVEC via caspase-dependent cell death pathway

**Figure 5:**
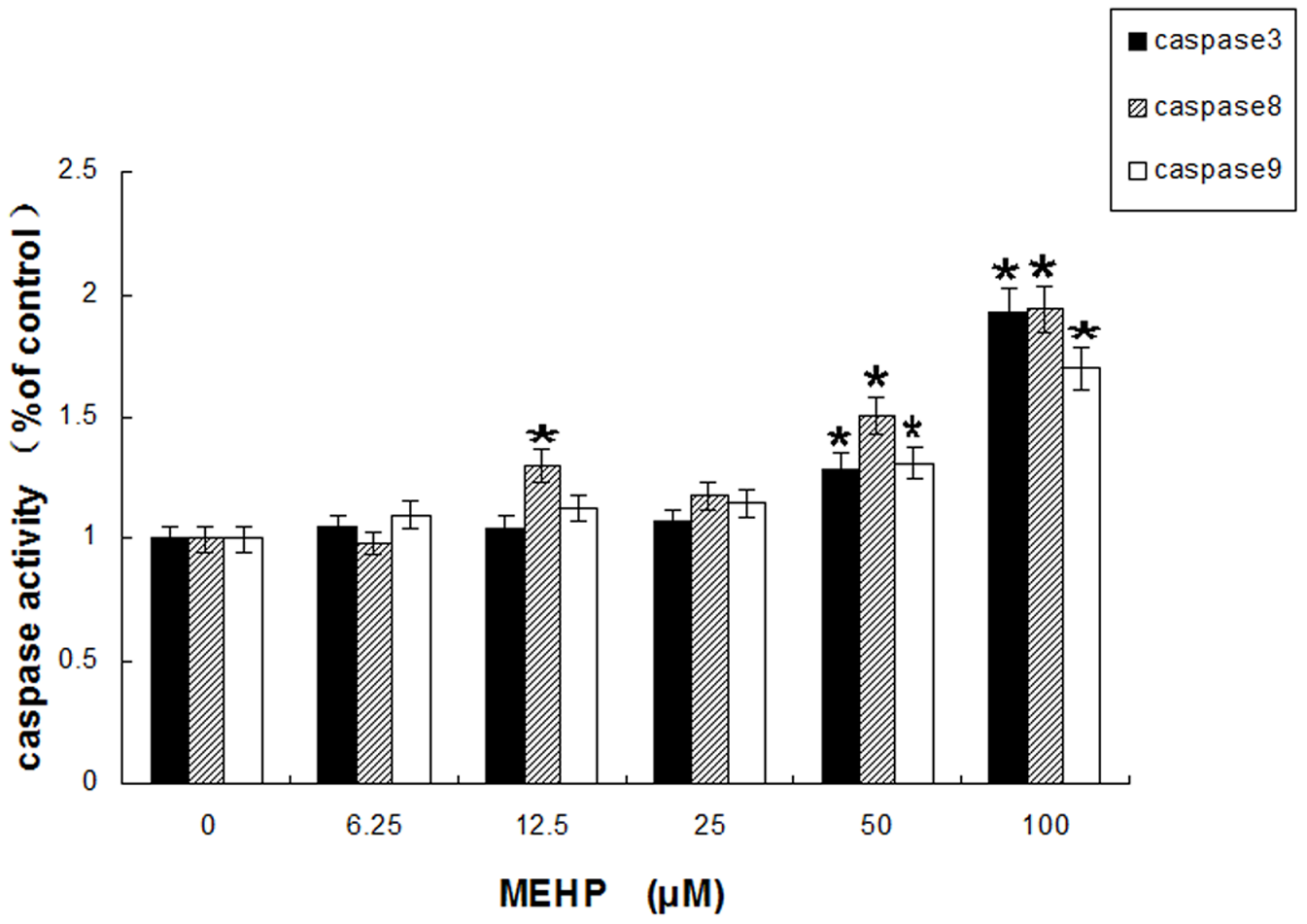
MEHP exposure (0, 6.25, 12.5, 25, 50 and 100 µM for 24 hours) affected enzyme activities of caspase-3, -8 and -9 in HUVEC cells. The data from three independent experiments presented in the form of means±SEM; n = 6. * P<0.05 was considered as statistically significant difference compared to control group.

It has been certificated that caspases cascade play a crucial role in cell apoptosis induced by multiple stimuli [16]. Decreased MMP initiate the process of cytochrome C releasing from mitochondria into the cytoplasm. Cytochrome C activates caspase-9 and then caspase-3, which leads to cell apoptosis. The caspase-like activity was measured by specific chromogenic substrates. As shown in Figure 5, after MEHP treatment (0, 6.25, 12.5, 25, 50 and 100 µM) for 24 hours, the activities of caspase-3, -9 and -8 were increased in a dose-dependently.

### Expression of Cytochrome C, Bax and Bcl-2

**Figure 6:**
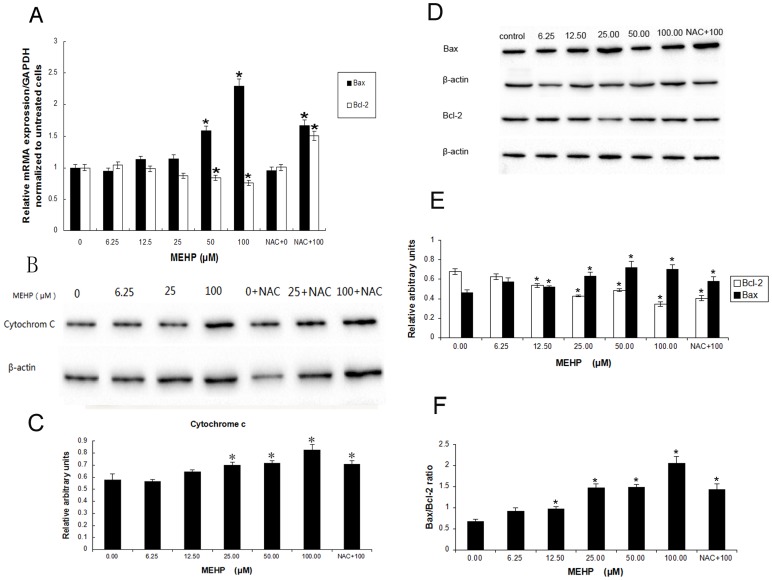
MEHP exposure affected mRNA and protein expression. (A) MEHP exposure affected Bax and Bcl-2 mRNA expression levels in HUVEC cells. The HUVEC cells were treated with 0, 6.25, 12.5, 25, 50, and 100 µM MEHP for 24 hours in MEHP treated group. In NAC+MEHP treated group, before MEHP administration the HUVEC cells was pretreated by NAC for 1 hour and then treated with 0 µM and 100 MEHP for 24 hours. The HUVEC cells in control group were only treated with 0.1% dimethyl sulfoxide (DMSO) for 24 hours. Bcl-2 and Bax mRNA expression was quantified after normalization to GAPDH. Data from three independent experiments were presented in the form of mean±SEM; n = 3. * P<0.05 was considered as statistically significant difference compared to the control group. (B,C,D,E,F) MEHP exposure affected cytochrome C, Bax and Bcl-2 protein expression in HUVEC cells. The HUVEC cells were treated with 0, 6.25, 12.5, 25, 50, and 100 µM MEHP for 24 hours in MEHP treated group. In NAC+MEHP treated group, before MEHP administration the HUVEC cells was pretreated by NAC for 1 hours and then treated with 0 µM and 100 MEHP for 24 hours. The HUVEC cells in control group were treated only with 0.1% dimethyl sulfoxide (DMSO) for 24 hours. Experiments were repeated in triplicate.Data presented are the mean±SEM from three independent experiments; n  = 6. * P<0.05, statistically significant difference compared with the control group.

To further certify whether MEHP exposure could change the expression of important pro-apoptotic and anti-apoptotic gene, we measured the mRNA expression of Bcl-2 and Bax by QPCR and the protein expression of Cytochrome C, Bax and Bcl-2 by western-blot in HUVEC cells after treatment with MEHP (0–100 µM). As shown in Fig. 6A, being normalized to GAPDH, the expression of Bcl-2 mRNA was decreased, while the expression of Bax mRNA was increased markedly corresponding to the MEHP dose. The ratio of Bax to Bcl-2 was increased significantly also in a dose-depended manner after MEHP administration. The Cytochrome C (Fig. 6B,C) releasing and Bax (Fig. 6D,E)protein expression increased in treatment group dose-dependently, whereas the Bcl-2 protein decreased (Fig. 6D,E), which is consistent with the mRNA expression.

### NAC Attenuates MEHP-induced Cell Apoptosis

As a thiol compound, NAC was considered to be a antioxidant for it regulates the redox status in cells and can act as a precursor of decreased glutathione and direct ROS scavenger[17]. In present study, NAC was applied to block ROS generation to investigate the role of ROS generation in MEHP-induced cell apoptosis. Figure 3(G, H) shows the levels of ROS generation in NAC pretreated HUVEC cells. It is obvious that compared to MEHP treated cells, the pretreated NAC attenuated the fluorescence in the microplate assay, which indicated that the pretreated NAC reduced ROS generation.

Furthermore, in the MTT assay the pretreated NAC induced increasing of cell viability from 58.5% in the MEHP treated cells to 85.0% in NAC prior MEHP treated cells, which indicated an obvious suppress effect on MEHP induced cytotoxicity (Fig. 1A). Pretreated NAC also showed similar effect MEHP induced cell apoptosis (Fig. 1B) and MMP loss (Fig. 4). Moreover, pretreated NAC induced remarkably increasing of Bcl-2 mRNA expression and reduction of the Bax/Bcl-2 ratio (Fig. 6A). Similar result was observed in the Bcl-2 protein expression (Fig. 6E).

## Discussion

Mono-(2-ethylhexyl) phthalate (MEHP), an active metabolite of di-(2-ethylhexyl) phthalate (DEHP), belongs to the phthalates family. It was reported that MEHP exposure may induce adverse effects in the reproductive system, increased carcinogenicity, and liver, kidney and developmental toxicities [10]–[12]. The previous study also showed maternal exposure to MEHP interfered nephron formation and induced hypertension in offspring [13]. Therefore, we infer that MEHP may have adverse effects on endothelial cells. In current study, it was demonstrated that low concentration of MEHP may induce oxidative stress and apoptosis in HUVEC cells in a dose-dependent manner via caspase-dependent pathway.

Increased reactive oxygen species (ROS) and/or reactive nitrogen species result in oxidative stress [18]. As mentioned in the results section, low concentration of MEHP (<100 µM) induced ROS generation in a dose-dependent manner by DCF-DA in HUVEC cells (Fig. 3). Antioxidant enzymes could alleviate the adverse effect s of oxidative stress, such as superoxide dismutase (SOD) and glutathione peroxidase (GPx). SOD attenuates oxidative stress by catalyzing the dismutation process, which converts the superoxide anion into molecular oxygen and hydrogen peroxide. It was indicated that GPx may detoxify hydrogen peroxides and lipid peroxides, and modulate redox-sensitive signaling pathways [19]. Malondialdehyde (MDA) is one of the end products of lipid peroxidation induced by ROS and free radicals and widely used to indicate cell injury [20]. As showed in Figure 2, MEHP in low concentration could increase MDA levels and SOD activity and decrease the GSH levels in a dose-dependent manner, indicating that the low dose of MEHP could induce cytotoxic effect in HUVEC cells. It was reported that increased lipid peroxidation in cell membrane may initiate gene expression and thereby cell proliferation, or apoptosis [21]. The MTT assay and PI staining demonstrates low dose MEHP represses cell viability and induces cell apoptosis in HUVEC in a dose-dependent manner (Fig. 1).

Apoptosis is defined as a energy dependent process of programmed cell death [22]. As the major ATP and hydrogen peroxide producer, mitochondrion is essential in initiating apoptosis [23]. Hydrogen peroxide may induce increased mitochondrial permeability, mitochondrial membrane potential disruption and releasing of cytochrome C from mitochondria into the cytoplasm, and therefore initiate apoptosis by activating caspase-3 [24]. Moreover, DNAs located in both nucleus and mitochondria may be involved in this oxidative damage. Previous studies indicated that in the liver of PPAR-alpha null mice DEHP induced 8-Hydroxy-20-deoxyguanosine (8-OHdG) formation, which is a major form of oxidative DNA injury induced by free radical [25]. It has been demonstrated that MEHP also induced oxidative stress and lipid peroxidation [26], [27]. It also has been reported that the mitochondrion membrane potential loss may be contributed to mitochondrion dysfunction and further cell apoptosis [16]. In the present study, we have demonstrated that low concentration of MEHP induced loss of MMP in a dose-dependent manner by detecting JC-1 fluorescence (Fig. 4).

As important regulators of apoptosis, the Bcl-2 family proteins are categorized into several groups by its function. One category is anti-apoptotic proteins which contain a transmembrane region and multiple Bcl-2 homology (BH) domains, such as Bcl-2 and Bcl-XL. Another category is proapoptotic proteins: Bax, Bak etc. There is also a rest category called proapoptotic ligands which contain only the BH3 domain, for instance, PUMA, NOXA, Bim, Bid etc. [28]. Activated Bax results in MMP loss, and induces the apoptotic proteins, such as cytochrome C and Smac/DIABLO, release from mitochondria into the cytoplasm. Cooperating with Apaf-1, the cytochrome C released into cytoplasm may activate caspase-9 and therefore induce activation of caspase cascade, including caspases-3, -6 and -7, resulting in cell apoptosis [29]. The Bax/bcl-2 ratio is pivotal in this process because it regulates cytochrome C release from mitochondria into cytoplasm and therefore determines whether cells suffer apoptosis [[30], [31], [32]]. As showed in Figure 5, the present study demonstrates that low dose of MEHP induced enhanced activity of caspase-3, -8 and -9 in HUVEC cells, indicating that low MEHP could active not only intrinsic but also extrinsic cell death pathway. It was also demonstrated that low concentration of MEHP induced Bax expression and Cytochrome C release from mitochondria to cytoplasm while reduced the expression of Bcl-2 at both transcription and protein translation level. More important, the Bax/bcl-2 ratio increased (Fig. 6).

NAC, a thiol compound, is considered to be a reduced glutathione precursor of and acts as a direct ROS scavenger [33]. In the current study, we also found that pretreated NAC before exposed to MEHP reduced intracellular ROS generation and the cytotoxicity results from MEHP in HUVEC cells. Moreover, pretreated NAC also attenuated MEHP-induced MMP decrease. Before MEHP administration, the pretreated NAC resulted in down-regulation of Bcl-2 mRNA expression and reduction of Bax/bcl-2 ratio, which might lead to an elevated level of Bcl-2 homodimers generation. The increase of Bcl-2 homodimners can prevent the MMP loss, suppress mitochondrial cytochrome C release to cytoplasm and block activation of the caspase cascade [34].

The previous works in our laboratory indicated that maternal DEHP exposure induced renal glomerulus injury in offspring. However, the potential mechanism was not mentioned. In the present study, it was demonstrated that MEHP could induce cytotoxic effect in endothelial cells via caspase-dependent cell death pathways, which may explain the mechanism of our previous works.

In summary, the present study investigated whether and how low concentration of MEHP could induce apoptosis in HUVEC cells. Meanwhile, the MEHP exposure in HUVEC cells induced intracellular ROS generation and result in loss of the mitochondrion membrane potential. Moreover, pretreated NAC in HUVEC cells attenuated oxidative stress and mitochondrion membrane potential disrupt induced by MEHP. As mentioned above, we infer that MEHP may induce HUVEC cell apoptosis via a mitochondrion-dependent pathway mediated by ROS.
